# Effect of Synthesis Method on Reaction Mechanism for Hydrogen Evolution over Cu_x_O_y_/TiO_2_ Photocatalysts: A Kinetic Analysis

**DOI:** 10.3390/ijms24032004

**Published:** 2023-01-19

**Authors:** Laura Clarizia, Giuseppe Vitiello, Robert Bericat Vadell, Jacinto Sá, Raffaele Marotta, Ilaria Di Somma, Roberto Andreozzi, Giuseppina Luciani

**Affiliations:** 1 Department of Chemical, Materials and Industrial Production Engineering, University of Naples “Federico II”, p.le V. Tecchio 80, 80125 Napoli, Italy; 2 CSGI, Center for Colloid and Interface Science, via della Lastruccia 3, 50019 Sesto Fiorentino, Italy; 3 Department of Chemistry-Ångström, Physical Chemistry Division, Uppsala University, P.O. Box 532, 751 20 Uppsala, Sweden; 4 Institute of Physical Chemistry, Polish Academy of Sciences, Kasprzaka 44/52, 01-224 Warsaw, Poland; 5 Istituto di Scienze e Tecnologie per l’Energia e la Mobilità Sostenibili (STEMS)-Consiglio Nazionale delle Ricerche, p.le V. Tecchio 80, 80125 Napoli, Italy

**Keywords:** photocatalysis, hydrogen production, kinetic modeling, Cu_x_O_y_, TiO_2_, Ohmic junction, long-lived charge-separated state, charge carrier recombination

## Abstract

The existing literature survey reports rare and conflicting studies on the effect of the preparation method of metal-based semiconductor photocatalysts on structural/morphological features, electronic properties, and kinetics regulating the photocatalytic H_2_ generation reaction. In this investigation, we compare the different copper/titania-based photocatalysts for H_2_ generation synthesized via distinct methods (i.e., photodeposition and impregnation). Our study aims to establish a stringent correlation between physicochemical/electronic properties and photocatalytic performances for H_2_ generation based on material characterization and kinetic modeling of the experimental outcomes. Estimating unknown kinetic parameters, such as charge recombination rate and quantum yield, suggests a mechanism regulating charge carrier lifetime depending on copper distribution on the TiO_2_ surface. We demonstrate that H_2_ generation photoefficiency recorded over impregnated Cu_x_O_y_/TiO_2_ is related to an even distribution of Cu(0)/Cu(I) on TiO_2_, and the formation of an Ohmic junction concertedly extended charge carrier lifetime and separation. The outcomes of the kinetic analysis and the related modeling investigation underpin photocatalyst physicochemical and electronic properties. Overall, the present study lays the groundwork for the future design of metal-based semiconductor photocatalysts with high photoefficiencies for H_2_ evolution.

## 1. Introduction

The need for renewable energy sources with low environmental impact has raised the research community’s interest in recent years. Hydrogen gas, as a green energy carrier, can be produced via sustainable processes employing unlimited energy sources, such as light irradiation [1]. Photocatalytic reforming of organics is a promising strategy for clean and low-cost light-driven hydrogen production. Photocatalytic reforming of organic species can compete with and even replace established processes based on the thermal processing of fossil fuels with high costs and severe operating conditions [1]. Nanocrystalline TiO_2_ in P25 form (i.e., 80:20 *w*/*w* anatase:rutile) is one of the most effective employed photocatalysts. However, it works under near-UV irradiation and exhibits a high likelihood of photogenerated charge carrier recombination [2,3,4]. The addition of copper species is considered an effective strategy to enhance P25-TiO_2_ photoefficiency and extend its light absorption range due to forming a metal–semiconductor heterojunction spatially separating free charge carriers [5,6].

Although several studies investigated the effect of copper loading on TiO_2_ nanoparticles (TiO_2__NPs) for the photocatalytic H_2_ evolution reaction (HER) [1], the role of copper oxidation state on the improvement in photocatalytic activity still needs to be clarified [7]. Conflicting hypotheses were developed in the literature survey to explain the improved photocatalytic activity of copper/P25-TiO_2_ catalysts. For instance, the presence of (i) finely dispersed and easily reducible cupric and cuprous oxides [7], (ii) Cu(0) [8], (iii) Cu_2_O [9,10,11], or (iv) CuO nanoparticles [12,13,14,15,16,17,18] on the TiO_2_ surface are reported among the possible vital factors affecting HER. Such widespread variability can be related to the complex structural, morphological, and chemical composition of Cu_x_O_y_/TiO_2_ photoactive materials [13,17]. As a result, copper-based species on the TiO_2_ surface may exist as a combination of mixed oxidation states, namely, Cu(0), Cu(I), and Cu(II). It is noteworthy that copper oxidation states can also vary during the photocatalytic process [5,19,20,21]. Previous findings established that both Cu(0) and Cu(I) resulting from Cu(II) reduction act as co-catalysts for photocatalytic H_2_ production [5,19,21]. Moreover, Cu(0) and Cu(I) boost the photoefficiency for HER during organic photoreforming via different reaction mechanisms, thus increasing past discrepancies in the behavior of these materials [5,19,21].

The synthesis procedure is crucial in modulating the physicochemical properties of Cu_x_O_y_/TiO_2_ photocatalysts. Indeed, the adopted photocatalyst preparation method defines copper oxidation state, particle size, and morphology, thus affecting photocatalytic H_2_ generation. Different techniques, such as hydrothermal/solvothermal processes, impregnation [22,23], precipitation [24], electrodeposition [25], and sol–gel methods [26], were used to dope TiO_2_ photoactive materials with copper species. H_2_ production through in situ photodeposited copper-TiO_2_ nanoparticles was investigated in previous studies. Notably, the photodeposition of Cu(0) nanoparticles on the P25-TiO_2_ surface dramatically affects the light absorption properties of titanium dioxide due to plasmon resonance phenomena. Furthermore, the photodeposition of Cu(0) on P25-TiO_2_ leads to distinct oxidation mechanisms of organic species in the aqueous phase depending on their tendency to adsorb on the catalyst surface [19].

Several examples of Cu_x_O_y_/TiO_2_ photocatalysts prepared via an impregnation/calcination method were reported recently [11,12,14,15,16,20,27,28,29,30,31,32,33,34,35,36,37]. It was demonstrated that Cu_x_O_y_ nanoparticles adsorbed on the TiO_2_ surface are involved in dissolution–reduction phenomena during the photocatalytic process, thus leading to the presence of both Cu_2_O and Cu(0) on the P25-TiO_2_ surface [21]. Both active copper species accounted for a remarkably improved photoefficiency for HER of copper-doped P25-TiO_2_ nanoparticles [21].

In this scenario, the present paper compares the effect of different Cu-TiO_2_-based photocatalyst synthesis procedures (i.e., impregnation/calcination and in situ photodeposition) on H_2_ evolution reaction. We demonstrate, for the first time, that in-depth kinetic modeling of the experimental results allows us to relate the photocatalytic performances for HER of the different materials to their unique physicochemical properties. The kinetic analysis and the associated mathematical model enable the evaluation of crucial unknown kinetic parameters regulating HER, such as the recombination rate of photogenerated carriers and the primary quantum yield. The estimated kinetic parameters strictly depend on metal-based semiconductor photocatalysts’ structural, morphological, compositional, and electronic features.

Overall, new insights into the reaction mechanism for HER of copper-TiO_2_ photocatalysts are provided by our investigation. Comprehensive information regarding charge carrier photogeneration and lifetime is reported for the first time. As a result, our investigation offers a new perspective for an effective design of metal-based nanophotocatalysts with remarkable photocatalytic H_2_ production.

## 2. Results and Discussion

### 2.1. Physicochemical Properties of Copper/P25-TiO_2_ Nanoparticles

The chemical, structural, morphological, and optical properties of the photocatalysts employed for the present investigation were previously reported in detail [5,19,21]. Material characterization indicates that copper-TiO_2_-based photocatalysts prepared via in situ photodeposition and impregnation/calcination method exhibit different copper nanoparticle distribution on the P25-TiO_2_ surface.

Table 1 reports an immediate view of the physicochemical characterization carried out for the photocatalysts investigated. Negligible differences in particle size and surface area of the two Cu-based photocatalytic materials were observed. Cu_x_O_y_/TiO_2__NPs synthesized via impregnation/calcination method have a slightly lower BET surface area compared with Cu/TiO_2__NPs prepared via photodeposition (i.e., 48.5 m^2^/g). Additionally, Cu_x_O_y_/TiO_2__NPs exhibit slightly smaller average sizes of copper and titania nanocrystals, as evidenced by XRD and HR-TEM analyses (see Table 1).

As regards copper oxidation state, Cu(0) and Cu_2_O species were detected via a combination of XRD, Raman, HR-TEM, EPR, and XPS analyses in Cu_x_O_y_/TiO_2__NPs prepared through impregnation/calcination. On the other hand, the sole presence of Cu(0) in Cu/TiO_2__NPs prepared via photodeposition was revealed by XRD, Raman, HR-TEM, EPR, and XPS analyses [5,19,21].

**Figure 1:**
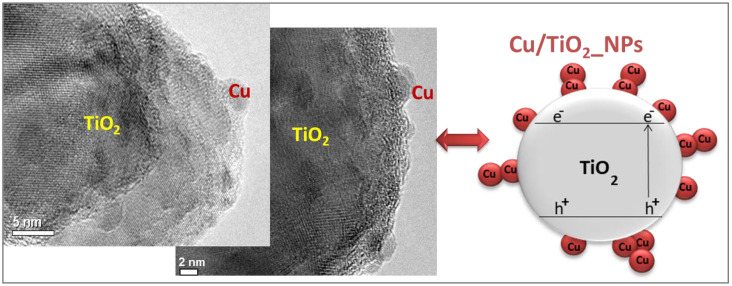
“Skyscraper distribution” of copper nanodeposits on the surface of in situ photodeposited Cu/TiO2_NPs evidenced by HR-TEM images.

The two different preparation methods induced a distinct arrangement of copper nanoparticles on the TiO_2_ surface. The in situ photodeposited Cu/TiO_2__NPs exhibit a non-homogeneous patchy distribution of metal copper on TiO_2_. According to experimental and theoretical data, metal nanoparticles are preferentially deposited over oxygen vacancies, acting as nucleating centers on the TiO_2_ surface [5,38]. The “skyscraper distribution” of metal copper on TiO_2_ is supported by HR-TEM images of Cu/TiO_2__NPs in Figure 1 [5,21]. However, a deeper analysis of the HR-TEM images indicates differences in the crystalline grape fringes of Cu nanoclusters. These differences indicate that the presence of traces of non-metallic copper species in the photodeposited photocatalyst cannot be ruled out, probably due to fast oxidation processes occurring under ambient conditions.

**Figure 2:**
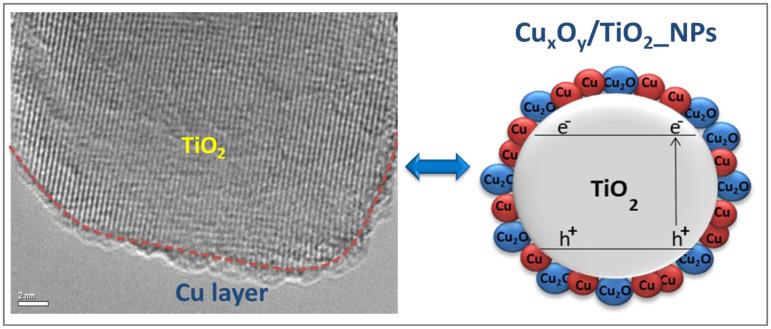
HR-TEM of Cu_x_O_y_/TiO_2__NPs prepared via impregnation/calcinations: even distribution of Cu_2_O and Cu(0) in a mixed copper state layer on P25-TiO_2_ (i.e., “core–shell” configuration).

In the case of impregnated Cu_x_O_y_/TiO_2__NPs, an even layer of Cu_x_O_y_ nanoparticles finely dispersed on P25-TiO_2_ was detected by morphological characterization, and is demonstrated in Figure 2. As shown in [21], Cu_x_O_y_ species undergo a dissolution–redeposition process leading to a uniform distribution of Cu_2_O and zero-valent copper on the TiO_2_ surface (i.e., “core–shell” configuration shown in Figure 2).

Photoluminescence analysis was also carried out in the present comparative study to understand the light emission behavior of the NPs. Figure S2A (Supplementary Material) shows the PL intensity for the samples. The PL signal centered at 435 nm is assigned to the radioactive recombination of trapped or bound electrons to the oxygen vacancy centers with valence holes on P25-TiO_2_ [39,40,41]. Adding Cu to the materials results in a significant quenching of the PL signal, which is related to electron transfer from P25-TiO_2_ to Cu(0) particles [42]. The Cu_2_O on Cu_x_O_y_/TiO_2__NPs bands in alignment with P25-TiO_2_ bands does not allow for electron transfer from P25-TiO_2_ to its conduction band [42]. There is also the possibility for hole transfer from P25-TiO_2_ valence to Cu_2_O valence, which would also decrease the PL yield. The PL signal suggests an increase in charge separation lifetime due to electron transfer from TiO_2_ to metallic copper species. Figure S2B (Supplementary Material) shows that the Cu_x_O_y_/TiO_2__NPs are slightly more effective in quenching the PL signal than Cu/TiO_2__NPs, consistent with electrons and holes being transferred from P25-TiO_2_.

### 2.2. Phenomenology of Typical Photoreforming Runs

**Figure 3:**
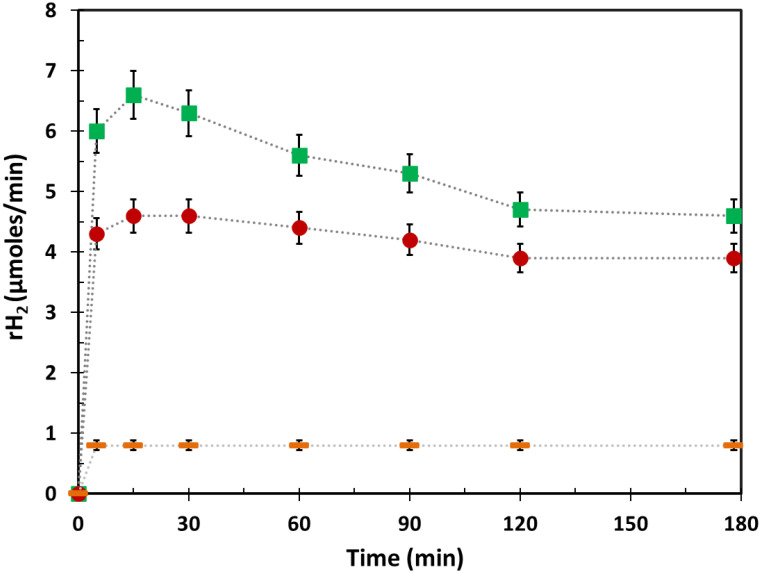
Hydrogen production rates with relative error bars during methanol photoreforming under de-aerated conditions in the presence of Cu/TiO_2__NPs prepared via photodeposition (⬤), Cu_x_O_y_/TiO_2__NPs prepared via impregnation method (■), and bare P25-TiO_2_ (**—**). Copper/TiO_2_ weight ratio = 3%; [CH_3_OH]_0_ = 2.47 M; photocatalyst load = 150 mg·L^−^^1^; T = 25 °C; P = 1 atm.

Figure 3 reports the hydrogen production rates (rH_2_) of a typical methanol photoreforming run over in situ photodeposited Cu/TiO_2__NP and impregnated Cu_x_O_y_/TiO_2__NP photocatalysts. By comparing hydrogen production rates under the same operating conditions (i.e., photocatalyst load, copper content, organic concentration, light irradiation wavelength, operating temperature and pressure, etc.), it appears that both materials exhibit an overshoot at around 15 min of light irradiation, after which a plateau value at 120 min of reaction is reached for both materials. It is noteworthy that the irradiance values recorded in both the UV and the visible range decrease within 15 min of reaction time (Figure S3, Supplementary Material), thus indicating higher light absorption of both Cu/TiO_2__NPs and Cu_x_O_y_/TiO_2__NPs with respect to TiO_2_-P25. The increase in light absorption of Cu/TiO_2__NPs and Cu_x_O_y_/TiO_2__NPs can be related to the formation of different active copper species, as explained below.

Table 2 reports the plateau values of rH_2_, and the associated error ranges recorded at different copper contents for both materials. A similar plateau value of rH_2_ can be observed at 3 wt.% and 6 wt.% of copper/P25-TiO_2_ for both Cu/TiO_2__NPs and Cu_x_O_y_/TiO_2__NPs. Such a comparison enables to the adoption of an optimum copper/P25-TiO_2_ weight ratio of 3% for both Cu/TiO_2__NPs and Cu_x_O_y_/TiO_2__NPs.

The solution pH had a constant value of about 6.5 throughout the experiment in the presence of Cu_x_O_y_/TiO_2__NPs. In this case, the solution color turns from light teal, typical of suspended TiO_2_ nanoparticles, to deep indigo, thus suggesting that a change in copper oxidation occurs during the photoreforming process (Table 2) [19]. Indeed, the material characterization (i.e., XPS, Raman, EPR, HR-TEM, and XRD analyses) confirms that Cu_x_O_y_ nanostructures initially present on the P25-TiO_2_ surface undergo a dissolution process upon light irradiation, followed by the reduction of Cu(II) ions to Cu(I) and Cu(0) by photogenerated electrons (reactions R1 and R2). This change in the copper oxidation state accounts for the reacting mixture’s different color and optical properties [21].
Cu(II) +e^−^ → Cu(I)E° (Cu(II)/Cu(I)) = 0.16 V(R1)Cu(I) + e^−^ → Cu(0)E° (Cu(I)/Cu(0)) = 0.52 V(R2)

On the other hand, a decrease in solution pH from 6.4 to 2.9 was observed after introducing copper in the TiO_2_-P25 suspension to directly obtain in situ photodeposited Cu/TiO_2__NPs [5,19]. After the photodeposition process, the solution pH was adjusted to a value of 6.5 for a proper comparison with the performances of Cu_x_O_y_/TiO_2__NPs. For in situ photodeposited Cu/TiO_2__NPs, a marked change in solution color from white, typical of P25-TiO_2__NPs, to purple was observed, thus indicating that chemical transformations of copper species occur during the photocatalytic experiment (Table 2). The compositional characterization (i.e., Raman, EPR, HR-TEM, and XRD analyses) confirms that cupric ions quickly reduce to metal copper, which smoothly deposits on the P25-TiO_2_ surface under UV/vis light irradiation [5,19].

### 2.3. Effect of Catalyst Load

**Figure 4:**
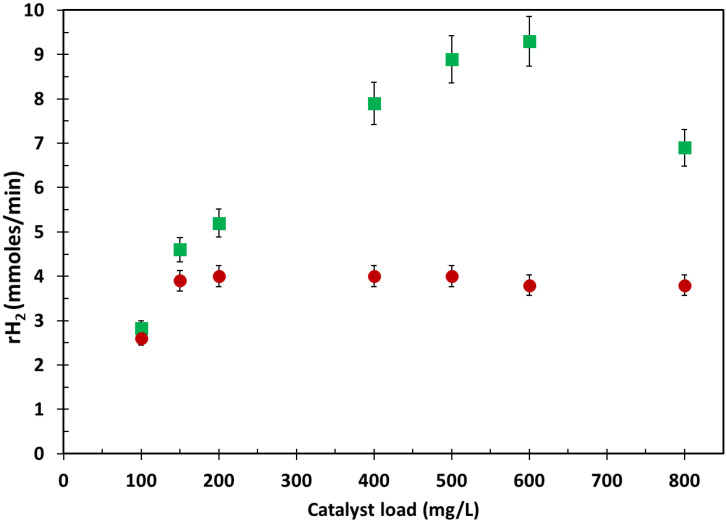
Plateau values of hydrogen production rates obtained during methanol photoreforming under de-aerated conditions in the presence of both photocatalytic materials at varying photocatalyst load ([CH_3_OH]_0_ = 2.47 M; copper:P25-TiO_2_ weight ratio = 3%; T = 25 °C; P = 1 atm). (■) Cu_x_O_y_/TiO_2__NPs; (⬤) Cu/TiO_2__NPs.

The effect of the catalyst load in the range of 100–800 mg/L was investigated for Cu_x_O_y_/TiO_2__NPs and Cu/TiO_2_ _NPs. A constant copper/P25-TiO_2_ mass ratio (i.e., 3 wt. %) was adopted. Figure 4 reports the hydrogen generation rates obtained at different catalyst loads for both materials. Hydrogen generation rates steadily rise by enhancing the photocatalyst load in 100–600 mg/L over Cu_x_O_y_/TiO_2__NPs prepared via impregnation. After reaching optimum hydrogen production of over 600 mg/L of CuxOy/TiO2_NPs, rH2 slightly reduces over higher catalyst loads (i.e., 800 mg/L), which is detrimental. This unfavorable effect is related to ineffective use of the incident UV/vis light irradiation due to light scattering and aggregation phenomena of photocatalyst nanoparticles [24,25].

A different trend is observed for the in situ photodeposited Cu/TiO_2__NPs. As shown in Figure 4, hydrogen generation increases linearly with low increases in catalyst load consistently with heterogeneous catalytic processes. A maximum increase in hydrogen generation is observed over a Cu/TiO_2__NP load equal to 150 ppm. Further increases in Cu/TiO_2__NPs amounts do not benefit the process photoefficiency.

### 2.4. Photocatalyst Reusability

**Figure 5:**
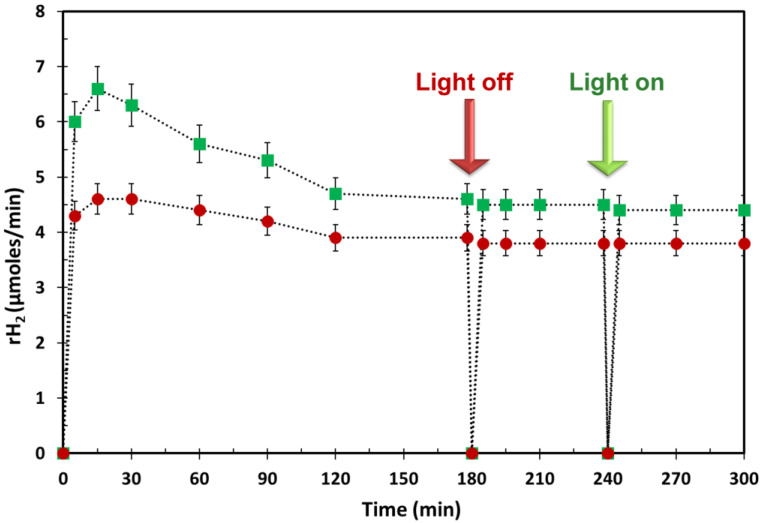
Hydrogen production rates and relative error bars during photocatalyst reusability in the presence of (■) Cu_x_O_y_/TiO_2__NPs prepared via impregnation method and (⬤) in situ photodeposited Cu/TiO_2__NPs. Copper:P25-TiO_2_ weight ratio = 3%; [CH_3_OH]_0_ = 2.47 M; photocatalyst load = 150 mg·L^−1^; T = 25 °C; P = 1 atm.

Reusability of both Cu/TiO_2__NPs and Cu_x_O_y_/TiO_2__NPs was tested. Figure 5 shows the results of a typical reusability test of methanol photoreforming over Cu_x_O_y_/TiO_2__NPs. After approaching the steady-state in hydrogen generation at t = 180 min, the lamp was switched off and then switched on once again. The results collected after this procedure indicate that the hydrogen production rate immediately achieves the previous plateau value. The new switch-on time was assumed as the zero-time for the modeling investigation. A similar trend was observed over in situ photodeposited Cu/TiO_2__NPs under intermittent UV/vis light irradiation. In this case, lower values of hydrogen production rate were recorded in steady-state conditions [19].

In addition, reusability tests over both photocatalytic materials were performed. The photocatalytic materials were recovered after the photoreforming runs according to the procedure described in Section 3.4. For both Cu/TiO_2__NPs and Cu_x_O_y_/TiO_2_, rH_2_ values equal the respective plateau values observed during their first use were observed.

### 2.5. Kinetic Modeling

Photocatalytic processes are promoted by photoactive materials capable of (i) ensuring rapid charge carrier generation and (ii) hampering electron–hole recombination [26]. Therefore, it is important to clarify how photocatalyst morphology and structure affect electronic properties. To this aim, kinetic models capable of evaluating the kinetic parameters of the photoreforming process are needed. However, only a restricted number of studies in the literature survey provide suitable kinetic assessments for HER over metal-based semiconductor photocatalysts [43,44,45]. Amongst the most relevant kinetic parameters, the following should be considered: quantum yield, photogenerated charge carrier recombination kinetic constant, the equilibrium constant of organic absorption on the catalyst surface, and kinetic constant regulating organic reaction with photogenerated holes. The availability of the best estimated values for these kinetic parameters allows both (i) a deeper comprehension of the effect of the photocatalyst physicochemical properties on HER and (ii) the upgrade of photoreforming processes to large-scale trials (i.e., real solar photoreactors).

Herein, we perform a novel kinetic analysis of the experimental outcomes collected over Cu_x_O_y_/TiO_2__NPs. Furthermore, the kinetic outcomes of the modeling investigation are compared with kinetic data of hydrogen generation over in situ photodeposited Cu/TiO_2_ _NPs [44]. Crucial information on the effect of the photocatalyst preparation method on electronic properties (i.e., photogeneration of charge carriers and their lifetime) are obtained, for the first time, by comparing the kinetic parameters underpinning the different copper-based TiO_2_ photocatalytic systems.

After considering that a couple of charge carriers are photogenerated upon irradiation of Cu_x_O_y_/TiO_2__NPs (r_3_), a reaction network was designed as follows.

(R3) $$Cu_{x}O_{y}/TiO_{2}\_NPs\overset{\;\;\;\;\;\;h\nu \;\;\;\;\;\;\;}{\to }e^{-}+h^{+}$$

(R4) $$e^{-}+h^{+}\overset{\;\;\;\;k_{r}\;\;\;}{\to }heat\;and\;light$$

(R5) $$MeOH+{∴}^{*}\;\;\;⇄\;\;MeOH{\;}^{*}$$

(R6) $$MeOH^{*}+h^{+}\overset{\;\;k_{h^{+}}}{\to }\;\;MeOH^{•}{}^{*}+H^{+}$$

(R7) MeOH•*+H+→     h+/fast        ∴*+products+2H+

(R8) H++e−→     fast    H•→     H•    /fast    H2

(1) $$\mathrm{rate}\;\mathrm{law}:\;G=\frac{\Phi_{UV}}{V}I_{a,\;UV\;}$$

(2) $$\mathrm{rate}\;\mathrm{law}:\;\;k_{r}\left[h^{+}\right]\cdot \left[e^{-}\right]$$

(3) $$\left[MeOH^{*}\right]=\frac{C_{T}\cdot K_{ads}\cdot \left[MeOH\right]}{\left(1+K_{ads}\cdot \left[MeOH\right]\right)}$$

(4) $$\;\mathrm{rate}\;\mathrm{law}:\;k_{h^{+}}\left[h^{+}\right]\left[MeOH^{*}\right]\;$$

Reaction rate R3 was calculated with Equation (1) by multiplying the quantum yield in the UV-A range $\left(\Phi_{UV}\right)$ and the radiation powers absorbed by the catalyst suspension ($I_{a,\;UV}$), and dividing by the volume of irradiated solution.

Photogenerated electrons and holes can react via non-radiative and radiative processes (r_4_). Reaction R4 is ruled by a second-order rate law Equation (2), where $k_{r}$ is the rate constant of photogenerated electron–hole recombination.

Photogenerated holes can also oxidize methanol adsorbed on the photocatalyst surface ($MeOH^{*}$) via reactions R6–R7. Reaction R6 is the rate-determining step for organic consumption Equation (3).

The Langmuir-type model reported for the equilibrium R_5_ allows one to estimate $\left[MeOH^{*}\right]$. To this aim, the concentration of the active sites on the catalyst surface C_T_ (M) at a fixed catalyst load q (g⋅L^−1^) and the adsorption equilibrium constant $K_{ads}$ (M^−1^) are considered in Equation (3). C_T_ is estimated by multiplying q and N (mol⋅g^−1^), which accounts for the moles of active sites per unit mass of catalyst.

Lastly, photogenerated electrons reduce protons from methanol oxidation to produce hydrogen gas (R8).

Based on the above-reported reaction network, a mathematical model built on mass balance equations for all species was developed Equations (5)–(9).

(5) $$\frac{d\left[e^{-}\right]}{dt}=G_{UV}-k_{r}\left[h^{+}\right]\left[e^{-}\right]-2k_{h^{+}}\left[MeOH^{*}\right]\left[h^{+}\right]$$

(6) $$\frac{d\left[h^{+}\right]}{dt}=G_{UV}-k_{r}\left[h^{+}\right]\left[e^{-}\right]-2k_{h^{+}}\left[MeOH^{*}\right]\left[h^{+}\right]$$

(7) $$\frac{d\left[MeOH\right]}{dt}=-k_{h^{+}}\left[MeOH^{*}\right]\left[h^{+}\right]$$

(8) $$\frac{d\left[H_{2}\right]}{dt}=k_{h^{+}}\left[MeOH^{*}\right]\left[h^{+}\right]$$

where

(9) $$G_{UV}=\frac{\Phi_{UV}}{V}I_{a,\;UV}=\frac{\Phi_{UV}}{V}\underset{i}{\sum }I_{\lambda_{i}}^{0}\left(1-e^{\left(-2.3\cdot \mu \cdot \varepsilon_{UV}\cdot q\right)}\right)$$

$I_{\lambda_{i}}^{0}$, μ, and $\varepsilon_{\mathrm{UV}}$ reported in Equation (9) are the power emitted by the lamp, the light path length, and the extinction coefficient of the photocatalyst in the UV/A wavelength range, respectively.

$\varepsilon_{\mathrm{UV}}$ is employed in the Lambert–Beer-law-like Equation (9) for evaluating the radiation power absorbed by the suspension ($I_{a,\;UV})$. An average value of $\varepsilon_{\mathrm{UV}}$ in the UV wavelength range (318 M^−1^∙s^−1^) was obtained through experimental measurements of $I_{a,\;UV}$.

The concentration of each species may be evaluated by assigning suitable values to the kinetic parameters involved to numerically integrate Equations (5)–(8). The following starting conditions are considered:

$${\left[S\right]}_{t=0}=S_{0},{\left[h^{+}\right]}_{t=0}=0,\;{\left[e^{-}\right]}_{t=0}=0,\;\mathrm{and}\;{\left[H_{2}\right]}_{t=0}=0$$

Appropriate values of $\Phi_{UV},N,\;k_{r}$*,*$k_{h^{+}}$, and $K_{ads}$ should be provided to use the kinetic model. Suitable values previously estimated for HER over copper/P25-TiO_2_ are adopted for $K_{ads}$ (i.e., the equilibrium constant of methanol adsorption on the photocatalysts) and $k_{h^{+}}$ (i.e., rate constant of reaction between adsorbed methanol and photogenerated positive holes) [44]. More in detail, starting from the Langmuir-type model reported for the equilibrium Equation (3) and plotting the term $1/r_{H_{2}}\;$against the reciprocal of methanol concentration, a suited value of $K_{ads}$ for Cu/TiO_2__NPs was obtained by the slope of the linear trend observed. After directly estimating $K_{ads}\;$from the experimental data using the Langmuir–Hinshelwood-type model, an optimum value of $k_{h^{+}}$ was evaluated through the modeling investigation [44].

On the other hand, $\Phi_{UV}$, N, and $k_{r}$ are parameters specific to the Cu_x_O_y_/TiO_2__NP photocatalyst, and should be evaluated. To this purpose, the numerical solution of the mass balance Equations (5)–(8) were fitted to the results of the experimental tests performed over Cu_x_O_y_/TiO_2__NPs (see Table 3).

Specifically, an iterative optimization procedure minimizing the squared difference between predicted and measured hydrogen generation rates was implemented in Matlab to obtain optimum values and confidence intervals for $\Phi_{UV}$, *N*, and $k_{r}$. Further details on the iterative optimisation procedure adopted are reported in the SM. First attempt values based on a previous study on methanol photoreforming over in situ photodeposited Cu/TiO_2__NPs [44] were used to run the optimization procedure. The starting values of the optimization procedure are reported in Table 4.

**Figure 6:**
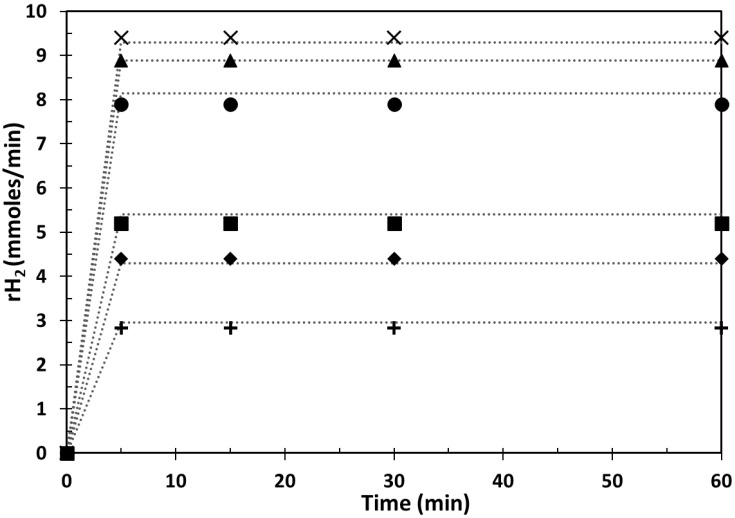
Comparison between experimental (symbols) and calculated values (dashed lines) for hydrogen generation rates recorded at different photocatalyst load over Cu_x_O_y_/TiO_2__NPs. [CH_3_OH]_0_ = 2.47 M; copper:P25-TiO_2_ weight ratio = 3%; T = 25 °C; P = 1 atm. Cu_x_O_y_/TiO_2__NP load: (✚) 100 mg∙L^−1^, σ = 4.5%. (♦) 150 mg∙L^−1^, σ = 2.2%. (■) 200 mg∙L^−1^, σ = 3.8%. (●) 400 mg∙L^−1^, σ = 3.1%. (▲) 500 mg∙L^−1^, σ = 0.1%. (✖) 600 mg∙L^−1^, σ = 1.1%.

Figure 6 shows a comparison between estimated and experimental data for hydrogen production through methanol photoreforming over impregnated Cu_x_O_y_/TiO_2__NPs at different photocatalyst load.

Table 5 reports the best estimated values of the unknown kinetic parameters resulting from the iterative optimization procedure. The widths of the confidence intervals reported are significantly lower than the optimal parameter values, thus evidencing a negligible uncertainty in the estimates.

By comparing the optimum kinetic parameters resulting from the modeling investigations on in situ photodeposited Cu/TiO_2__NPs and impregnated Cu_x_O_y_/TiO_2__NPs (see Table 5), novel information on the electronic properties and the mechanism of HER are obtained for the photocatalysts developed.

**Figure 7:**
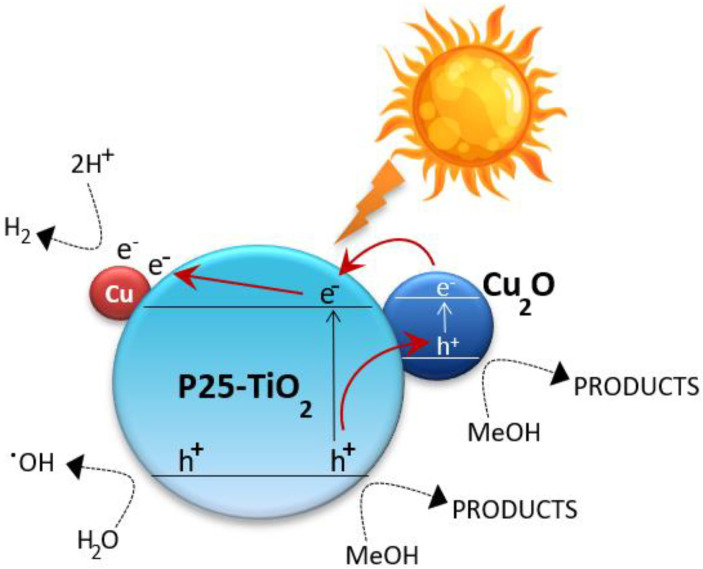
Mechanism of hydrogen production and methanol oxidation over Cu_x_O_y_/TiO_2__NPs.

As shown in Table 5, the best estimated value of the rate constant for electron–hole recombination (k_r_) of impregnated Cu_x_O_y_/TiO_2__NPs is more than two orders of magnitude lower than in situ photodeposited Cu/TiO_2__NPs and six orders of magnitude lower than bare P25-TiO_2_. CuO and Cu_2_O are obtained on the surface of Cu_x_O_y_/TiO_2__NPs prepared by impregnation and further heat treatment in nitrogen. Cu_x_O_y_ species on Cu_x_O_y_/TiO_2__NPs undergo an in situ dynamic nanostructuring during the photocatalytic process. Indeed, a change in both size distribution and copper oxidation state was observed. As shown in Figure 7, Cu_2_O and zero-valent copper act as co-catalysts for HER on Cu_x_O_y_/TiO_2__NPs. Under UV/vis light irradiation, Cu_2_O injects photoelectrons into the P25-TiO_2_ conduction band. Cu(0) acts as a co-catalyst by accepting photogenerated electrons from TiO_2_ and mediating their migration to protons [15,46].

Both processes lower electron–hole recombination and increase HER photoefficiency of impregnated Cu_x_O_y_/TiO_2__NPs. Conversely, the sole Cu(0) nanodeposits found on in situ photodeposited Cu/TiO_2__NPs act as active trap centers for photogenerated electrons. Indeed, an Ohmic junction forms on Cu_x_O_y_/TiO_2__NPs due to the presence of metallic Cu nanoparticles between the p-and n-type semiconductors (i.e., Cu_2_O and P25-TiO_2_, respectively). As previously reported [48,49], HER is favored upon UV/vis light irradiation of Cu_2_O/Cu/TiO_2_ photocatalysts due to the following phenomena: (i) the relatively low resistance of metal Cu metal helps improve photoelectron transfer from excited Cu_2_O to P25-TiO_2_; (ii) metal Cu metal acts as an electron storage center and favors charge separation [2,19,21].

The optimum value obtained for the primary quantum yield ($\Phi_{UV}$, the ratio between the moles of photogenerated charge carriers per mole of absorbed photons [50]) should also be considered in the present analysis. $\Phi_{UV}$ has a lower optimum value (i.e., $\Phi_{UV}$ = 0.04) for impregnated Cu_x_O_y_/TiO_2__NPs to in situ photodeposited Cu/TiO_2__NPs (i.e., $\Phi_{UV}$ = 0.19) under equal wavelengths [25,44]. The higher $\Phi_{UV}$ value of in situ photodeposited Cu/TiO_2__NPs results in a remarkable charge carrier photogeneration, due to the direct access of UV/vis light irradiation in the Cu-free spots. Indeed, as previously discussed [5], and evidenced by Figure 1, the uneven “skyscraper” distribution of copper deposits on in situ photodeposited Cu/TiO_2__NPs leaves a substantial proportion of P25-TiO_2_ surface available to absorb incident light irradiation directly. This phenomenon results in a higher $\Phi_{UV}$ of the photocatalytic material. However, a more significant number of photogenerated electron–hole pairs per absorbed photons on the Cu-free P25-TiO_2_ surface indicates that it has a shorter lifetime and quicker recombination, as confirmed by PL analysis and lower hydrogen generation rates.

Despite the resulting lower concentration of photogenerated charge carriers per moles of adsorbed photons (i.e., $\Phi_{UV}$) of impregnated Cu_x_O_y_/TiO_2__NPs, both Cu_2_O and zero-valent copper act as co-catalysts for H_2_ generation. This phenomenon reduces electron–hole recombination (as proven by PL spectra) and lengthens the NP lifetime, thus accounting for higher hydrogen generation rates over Cu_x_O_y_/TiO_2__NPs. In addition, the value of the total moles of active sites per unit mass of catalyst (i.e., N = 6.098 × 10^−5^ mol g^−1^) estimated for Cu_x_O_y_/TiO_2__NPs is one order of magnitude smaller than in situ photodeposited Cu/TiO_2__NPs (i.e., N = 3.69 × 10^−4^ mol g^−1^). This modeling outcome indicates that copper impregnation significantly modifies the semiconductor surface. According to literature findings [51], the total moles of active sites per unit mass of catalyst (mol∙g^−1^) can be calculated through Equation (10), as follows:

(10) $$N=\frac{S_{A}}{N_{A}\cdot S_{sat}^{0}}$$

where

○ S_A_ is the surface area of the photocatalyst (see Table 1).
○ N_A_ is the Avogadro’s number.
○ S^0^_sat_ is the surface area of photocatalyst covered by one molecule of adsorbed methanol.

Using Equation (10), a value of P25-TiO_2_ surface area covered by one molecule of adsorbed methanol on Cu_x_O_y_/TiO_2__NPs (i.e., S^0^_sat_ = 1.08 × 0^−18^) significantly higher than in situ photodeposited Cu/TiO_2__NPs (i.e., S^0^_sat_ = 0.22 × 10^−18^) is estimated. To shed light on this result, it must be considered that oxygen vacancies act as active sites for organic oxidation. Indeed, organic molecules are adsorbed on oxygen vacancies and neighbor-bridging oxygen sites via proton transfer [52]. At the same time, both experimental data and theoretical calculations show that metal nanoparticles are preferentially located over oxygen vacancies, which act as nucleating centers on the TiO_2_ surface [38]. Therefore, a more uniform distribution of Cu_2_O and Cu(0) in a mixed copper layer (i.e., the “core–shell” configuration shown in Figure 2) on impregnated Cu_x_O_y_/TiO_2__NPs indicates a higher density of oxygen vacancies. These oxygen vacancies are evenly distributed on the TiO_2_ surface and ready to act as active sites for methanol oxidation. Thus, the greater value S^0^_sat_ of Cu_x_O_y_/TiO_2__NPs can be related to the increased density of oxygen vacancies proven by the more uniform copper distribution on the photocatalyst surface [5]. All these outcomes confirm that the higher quantity of finely dispersed copper deposits on Cu_x_O_y_/TiO_2__NPs is a crucial factor for increasing hydrogen generation [13,46,48,53,54,55]. Consequently, it is worthwhile to tune the preparation method and guarantee a homogeneous coverage of the TiO_2_ surface [27].

Additionally, the reliability of the mathematical model developed is proven by simulating the results of photoreforming runs not previously included in the optimization procedure. No further adjustments of the best estimated kinetic parameters were made (i.e., simulation mode of the mathematical model). For this purpose, data from photocatalytic runs carried out at different starting methanol concentrations were employed. Table 6 reports the values of the percentage standard deviation estimated by considering experimental and theoretical data on hydrogen generation rates. The low values of the percentage standard deviation estimated allow us to affirm that the model can affordably predict the system behavior at varying operating conditions.

### 2.6. Technical Feasibility

As a preliminary remark, both photocatalytic materials developed for our investigation are based on low-cost, broadly available, and non-toxic elements (i.e., copper and titanium dioxide).

As regards the synthesis procedure employed, in situ photodeposition is an easy and cheap method based on the use of the same radiation source for both photocatalyst preparation and hydrogen generation.

Regarding the phenomenology of photocatalytic runs, a decrease in solution pH was observed during methanol photoreforming over in situ photodeposited Cu/TiO_2__NPs, (see Section 2.2). This decrease in solution pH would requires the alkalinization of the resulting mixture, given real applications on an industrial scale. Conversely, a constant neutral pH of the reacting mixture is measured during photoreforming runs over impregnated Cu_x_O_y_/TiO_2__NPs.

The photoactivity of both photocatalysts was successfully tested under intermittent light irradiation. This experimental outcome allows us to exclude photocatalyst deactivation and prospect an efficient photocatalyst use upon discontinuous light sources (i.e., in real solar applications). Moreover, both photocatalysts recovered adequately through a simple procedure after their use and exhibited constant hydrogen production rates compared with their earliest use. This evidence further supports the possibility of efficient photocatalyst reusability for multiple photocatalytic applications with an overall cost reduction.

## 3. Materials and Methods

### 3.1. Materials

Methanol (99.8% *v/v*), TiO_2_ Aeroxide-P25 (80/20 anatase/rutile, CAS 13463-67-7, product number 718467), copper(II) oxide (CuO, purity 99.9%), and cupric nitrate (Cu(NO_3_)_2_⋅3H_2_O, purity 98%) were purchased from Sigma Aldrich (Burlington, MA, USA). Double-distilled water was used to prepare the reacting mixtures for photocatalytic experiments.

### 3.2. Photocatalysts Preparation

#### 3.2.1. Impregnated Cu_x_O_y_/TiO_2_

Impregnated photocatalysts loaded with various weight percentages of copper (i.e., 3–16 wt.%) were prepared by employing commercial bare P25-TiO_2__NPs through an impregnation method described elsewhere [21]. For each copper-modified P25-TiO_2_ sample (Cu_x_O_y_/TiO_2__NPs), 1 g of P25-TiO_2__NPs was dispersed in 250 mL of an aqueous solution. The aqueous solution contained a proper concentration of Cu(NO_3_)_2_⋅3H_2_O salt to obtain Cu weight percentages of 3, 6, 10, and 16. Excess water was evaporated at slow heating rates under continuous stirring. Each sample was dried at 110 °C and calcined under N_2_ atmosphere for 5 h at 350 °C.

#### 3.2.2. In Situ Photodeposited Copper/TiO_2_ Photocatalysts

In situ photodeposited copper/TiO_2_ photocatalysts (Cu/TiO_2__NPs) were obtained from commercial bare P25-TiO_2__NPs through an in situ preparation procedure [5,19]. This approach allows the simultaneous occurrence of light-induced deposition of copper species on the surface of P25-TiO_2__NPs and methanol photoreforming in an aqueous solution for H_2_ generation. A fixed amount of bare P25-TiO_2__NPs (50 ÷ 500 mg L^−1^) was initially suspended in an unbuffered aqueous solution in the presence of copper(II) oxide (i.e., the copper precursor) and methanol (i.e., the sacrificial agent).

The reacting mixture was gently evaporated under a nitrogen atmosphere at the end of the photocatalytic experiments. Then, Cu/TiO_2__NPs were washed multiple times with de-aerated double-distilled water and dried under a nitrogen atmosphere.

### 3.3. Photocatalyst Characterization

Physicochemical properties of the herein-employed copper-modified P25-TiO_2_ nanoparticles in terms of morphology, structure, optical features, and oxidation state of copper species were previously evaluated through a combined approach of several analytical techniques, as described in Section 3.3.1, Section 3.3.2 and Section 3.3.3. Complete results of the physicochemical material characterization are reported elsewhere [5,19,21].

#### 3.3.1. Structural and Morphological Characterization

Structural and compositional characterizations of the photocatalysts were performed by X-ray diffraction (XRD), Brunauer–Emmett–Teller (BET) N_2_ adsorption analysis, and high-resolution transmission electron microscopy (HR-TEM).

XRD measurements were performed to identify the crystalline properties of the nanocomposites on a PANalytical diffractometer with a nickel filter and Cu Kα radiation.

BET analysis allowed us to assess the specific surface area (SBET), estimated by generating seven-point isotherms at 77 K for N_2_ adsorption (Autosorb-1, Quantachrome (Boynton Beach, FL, USA)). A char sample capable of providing a specific surface area equal to 5 m^2^ in the sample cell was used as a reference.

Extensive information on the surface morphology of the photocatalysts (i.e., crystal structure and size) were obtained on a JEM-2010F (JEOL) high-resolution transmission electron microscope with a field emission gun at 200 kV. The samples were prepared for HR-TEM analysis by dispersing the obtained solids in acetone, employing an ultrasonicator, and finally fixed on a carbon-coated copper grid (FCF400-Cu, FROMVAR).

#### 3.3.2. Compositional Characterization

Raman spectra, X-ray Photoelectron Spectra, and EPR measurements of the two different copper-modified P25-TiO_2__NPs were previously performed and described in detail elsewhere [5,19,21].

#### 3.3.3. Photoluminescence Analysis

The optical properties of copper-modified TiO_2_ nanoparticles were investigated by photoluminescence (PL) analysis. Specifically, three different samples (bare P25-TiO_2__NPs, Cu/P25-TiO_2__NPs, and Cu_x_O_y_/P25-TiO_2__NPs) were suspended in 5 mL of a degassed (20 min in N_2_) aqueous solution with pH ≈ 4 (adjusted by addition of nitric acid solution). To prevent changes in the copper oxidation state, the transference of the powder to the keys was performed under a nitrogen atmosphere. To ensure a homogeneous suspension, the vials were sonicated for five minutes. Finally, 0.1 mL of each suspension was further diluted in 3 mL of degassed aqueous media at pH ≈ 4. UV-vis measurements (Cary 5000 UV-Vis-NIR) before and after PL measurements were performed to ensure that samples preserved their suspension integrity and that semiquantitative considerations about the PL signals can be drawn. PL measurements were performed in a Fluorolog-3 fluorometer from Horiba Jobin Yvon. The PL spectra were obtained at 335 nm excitation (i.e., the wavelength showing the best compromise between signal-to-noise and no Raman scattering). PL spectra intensity were corrected for sample absorbance at 335 nm to remove the influence of different sample concentrations in the suspensions.

### 3.4. Photocatalytic Experiments

Photocatalytic experiments of methanol photoreforming were performed in an annular glass batch reactor (V = 300 mL) cooled at 25 °C through a thermostatic bath (Falc GTR 90). Light irradiation was provided by a high-pressure mercury vapour lamp (Helios Italquartz, Cambiago, Italy, power input: 125 W), primarily emitting at 305 nm, 313 nm, and 366 nm. The effective radiative powers and the emission spectrum of the lamp are reported in detail in Table S1 and Figure S1 (Supplementary Material (SM)), respectively. A light path length of 1.1 cm was estimated. An inlet was used to feed reactants and gaseous nitrogen into the top of the photoreactor. An outlet was used to recover gaseous and liquid samples at fixed reaction times.

For the photocatalytic experiments, a proper amount of photocatalyst was suspended in the aqueous mixture (V = 300 mL) containing methanol ([MeOH] = 10% *v/v*). The solution pH was not changed. The photocatalyst was kept well dispersed in the solution via continuous magnetic stirring at 500 rpm. A nitrogen flow was fed (N_2_ flow rate = 0.3 L/min) for 30 min before each photoreforming experiment to avoid the parasitic reaction between dissolved oxygen and photogenerated electrons. The system was kept under a nitrogen atmosphere to prevent air inlets into the photoreactor during the photocatalytic runs. Gaseous samples were collected in Tedlar bags and promptly injected into the gas chromatograph to evaluate the H_2_ generation rate. Withdrawn liquid samples were quickly filtered on regenerated cellulose filters (pore diameter 0.45 μm, Scharlau). Filtrates were employed to estimate the total dissolved copper concentration through a spectrophotometric procedure reported in Section 3.5.

Irradiances emerging from the photocatalytic reactor were also evaluated in different wavelength ranges (i.e., 315–400 nm and 400–1100 nm) during methanol photoreforming. To evaluate the photocatalytic activity under visible light irradiation, water in the cooling jacket was replaced by a 1 M NaNO_2_ aqueous solution absorbing UV irradiation in selected experiments. As earlier reported [19,21], nominal hydrogen production rates were recorded in the presence of the UV cutoff solution. After the photoreforming runs, the slurry suspensions settled under the nitrogen atmosphere. Two distinct layers could be distinguished thereafter: aqueous solution and Cu-modified P25-TiO_2__NPs on the bottom. Then, the mixture was gently evaporated under a nitrogen flow. Solid nanoparticles were washed multiple times with de-aerated double-distilled water and ultimately dried under an inert atmosphere.

### 3.5. Analytical Methods

Hydrogen production was measured by analyzing gaseous samples in a gas chromatograph (Agilent 7820A) with a TCD detector. The detector employed argon as the carrier gas and an HP-PLOT Molesieve 5A column (Agilent).

The concentration of dissolved copper in liquid samples was estimated through a previously reported colorimetric method [19]. The solution pH was monitored using an Orion 420A pH meter (Thermo (Waltham, MA, USA)). Irradiance values were measured on the external walls of the photoreactor through a digital radiometer (Delta Ohm HD 2102.1 (Dentro, Italy)).

## 4. Conclusions

Cu_x_O_y_/TiO_2__NPs and Cu/TiO_2__NPs for photocatalytic hydrogen generation were synthesized via impregnation/calcination and in situ photodeposition, respectively. A detailed experimental and modeling comparison among two different copper/TiO_2_ photocatalysts based on structural, morphological, photocatalytic, and kinetic properties is proposed. In particular, the effect of the preparation method on (i) photoefficiency for hydrogen generation and (ii) the value of crucial kinetic parameters regulating HER are investigated.

In situ photodeposition and calcination/impregnation methods affect the electronic properties of the semiconductor photocatalysts in different ways.

The best estimates of the kinetic parameters from the modeling investigation on Cu_x_O_y_/TiO_2__NPs and Cu/TiO_2__NPs allow us to deduce the following remarks on the electronic structure and HER mechanism.

- The Ohmic junction formed by metallic Cu and Cu_2_O on impregnated Cu_x_O_y_/TiO_2__NPs promotes charge carrier separation. This accounts for PL quenching and a rate constant for electron–hole recombination (k_r_) lower than in situ photodeposited Cu/TiO_2__NPs. As a result, higher hydrogen generation rates are recorded over Cu_x_O_y_/TiO_2__NPs.
- The optimum value of the primary quantum yield for impregnated Cu_x_O_y_/TiO_2__NPs is lower than in situ photodeposited Cu/TiO_2__NPs. This kinetic outcome is related to the different copper distributions on the P25-TiO_2_ surface. Indeed, the even Cu(0)/Cu_2_O distribution in Cu_x_O_y_/TiO_2__NPs evidenced by the structural and morphological characterization acts as a soft shield for UV-visible light absorption, thus reducing the specific amount of photogenerated couples. At the same time, the Cu(0)/Cu_2_O/P25-TiO_2_ core–shell structure in Cu_x_O_y_/TiO_2__NPs significantly enhances photogenerated charge carrier lifetime, thus improving the overall process photoefficiency. Conversely, a “skyscraper” distribution of sole Cu(0) on the P25-TiO_2_ surface was detected by the structural/morphological characterization of in situ photodeposited Cu/TiO_2__NPs. Such a patchy Cu(0) distribution allows the direct absorption of incident UV/vis light irradiation on the P25-TiO_2_ surface in Cu-free spots, thus resulting in a higher specific amount of photogenerated charge carriers. This electronic phenomenon is evidenced by the higher $\Phi_{UV}$ value of Cu_x_O_y_/TiO_2__NPs.
- A more uniform distribution of copper species on TiO_2_, as well as the simultaneous presence of different active oxidation states regulating migration and transfer of photogenerated carriers, accounts for the improved photoefficiency of Cu_x_O_y_/TiO_2__NPs.
- The reusability of both photocatalysts was successfully tested. Therefore, the possibility of an efficient photocatalyst use in the presence of accurate intermittent light irradiation is proven. The in situ photodeposition method is an easy and low-cost photocatalyst preparation procedure that enables the use of the same radiation source for photocatalyst synthesis and hydrogen generation. However, the need for alkalinization of the final aqueous mixture after the photocatalytic process challenges its commercial viability, given actual implementations.

Overall, this study outlines the importance of properly tuning the photocatalyst preparation method to guarantee (i) a homogeneous coverage of copper on the titania surface and (ii) efficient use of incident light irradiation for HER. The kinetic investigation provides an exhaustive basis for comprehending the dependence of the photocatalyst electronic structure on the synthesis procedure employed. This study opens the way to new effective strategies for developing high-performance metal-based semiconductor photocatalysts for hydrogen generation.

## Figures and Tables

**Table 1:** Physicochemical features of Cu-TiO_2_-based photocatalysts developed [5,19,21], (see SM herein provided).

Sample (Preparation Method)	Physicochemical Property Identified/Characterization Technique Employed				
	Surface Area (m^2^/g)	Titania Crystalline Phase	Average Titania Nanoparticle Size (nm)	Average Cu Nanoparticle Size (nm)	Active Cu Species
Cu/TiO_2_ _NPs (photodeposition)	48.5 BET	80% anatase, 20% rutile XRD	30.0 XRD, HR-TEM	4.0 HR-TEM	Cu(0) XRD, HR-TEM, XPS, Raman, EPR
Cu_x_O_y_/TiO_2_ _NPs (impregnation)	41.0 BET	80% anatase, 20% rutile XRD	25.0 XRD, HR-TEM	3.0 HR-TEM	Cu(0); Cu_2_O XRD, HR-TEM, XPS, Raman, EPR

**Table 2:** Plateau values of hydrogen production rates during methanol photoreforming under de-aerated conditions in the presence of Cu-modified P25-TiO_2_ at varying copper/TiO_2_ weight ratio ([CH_3_OH]_0_= 2.47 M; TiO_2_ load = 150 mg·L^−^^1^; T = 25 °C; P = 1 atm). Starting appearance of the reacting mixtures containing 3 wt.% copper/P25-TiO_2_ prepared via photodeposition (Cu/TiO_2__NPs) or impregnation (Cu_x_O_y_/TiO_2__NPs). The appearance of the same reacting mixtures after 180 min of photocatalytic run.

	Sample (Preparation Method)	Copper/P25-TiO_2_ Weight Ratio (%)				Starting Appearance	Final Appearance
		3	6	10	16		
**r_H2_ (μmol·min^−1^)**	Cu/TiO_2__NPs (photodeposition)	3.9 ± 0.2	4.2 ± 0.5	4.5 ± 0.3	2.8 ± 0.2	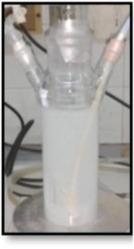	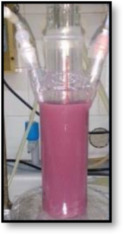
	Cu_x_O_y_/TiO_2__NPs (impregnation)	4.6 ± 0.3	5.2 ± 0.3	3.4 ± 0.2	2.1 ± 0.1	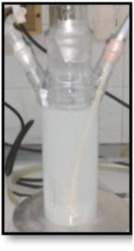	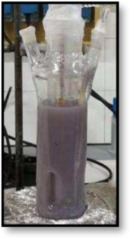

**Table 3:** Operating conditions of the photocatalytic runs over impregnated Cu_x_O_y_/TiO_2__NPs employed in the optimization procedure.

Run	[Cu]/[TiO_2_] (Wt. %)	T (°C)	Cu/TiO_2_-P25 Load (mg∙L^−1^)	[MeOH]_0_ (M)
I	3	25	100	2.47
II	3	25	150	2.47
III	3	25	200	2.47
IV	3	25	400	2.47
V	3	25	500	2.47
VI	3	25	600	2.47

**Table 4:** First attempt values for the kinetic parameters involved in the mathematic model [25].

Parameter	Value	Unit	To be Optimized
$k_{h^{+}}$	1.13 × 10^4^	M^−1^s^−1^	No
$K_{ads}$	0.24	M^−1^	No
$k_{r}$	3.91 × 10^6^	M^−1^s^−1^	Yes
N	3.69 × 10^−4^	mol∙g^−1^	Yes
$\Phi_{UV}$	0.19	mol∙E^−1^	Yes

**Table 5:** Best estimates of the unknown kinetic parameters.

Parameter	Unit	Best Estimated Value for Impregnated Cu_x_O_y_/TiO_2_-P25	Reported Value for in situ Photodeposited Cu/TiO_2_-P25_NPs [44]	Reported Value for Bare TiO_2_ [44,47]
$k_{r}$	M^−1^∙s^−1^	1.063 × 10^4^ ± 4.367 × 10^2^	3.91 × 10^6^	3.00 × 10^10^
N	mol∙g^−1^	6.098 × 10^−5^ ± 5.123 × 10^−8^	3.69 × 10^−4^	3.98 × 10^−4^
$\Phi_{UV}$	mol∙E^−1^	0.045 ± 0.001	0.19	0.06

**Table 6:** Percentage standard deviation and plateau value of hydrogen generation rates predicted by the kinetic model and recorded over a photocatalytic run at different starting methanol concentrations. Copper:P25-TiO_2_ weight ratio = 3%; T = 25 °C; P = 1 atm.

Run	[Cu]/[TiO_2_] (Wt. %)	T (°C)	Cu_x_O_y_/P25-TiO_2_ Load (mg∙L^−1^)	[MeOH]_0_ (M)	r_H2_ Calculated at t = 60 min (μmoles/min)	r_H2_ Measured at t = 60 min (μmoles/min)	σ_RUN_ (%)
VII	3	25	150	1.64	3.53	3.56	0.85
VIII	3	25	150	0.82	2.31	2.40	3.80
IX	3	25	150	0.41	1.36	1.41	3.67

## Data Availability

Not applicable.

## Supplementary Materials

The following supporting information can be downloaded at https://www.mdpi.com/article/10.3390/ijms24032004/s1.
