# Supplementary material for: Effect of Synthesis Method on Reaction Mechanism for Hydrogen Evolution over CuxOy/TiO2 Photocatalysts: A Kinetic Analysis
Source: Int J Mol Sci. 2023 Jan 19;24(3):2004. doi: 10.3390/ijms24032004 (PMC9916258; doi:10.3390/ijms24032004)
Supplement: Supplementary file 1 [file ijms-24-02004-s001.zip › ijms-2123568-supplementary.pdf]

## Supplementary information

# Effect of synthesis method on reaction mechanism for hydrogen evolution over $Cu_xO_y/TiO_2$ photocatalysts: a kinetic analysis

**Laura Clarizia [1,*], Giuseppe Vitiello [1,2,*], Robert Bericat Vadell [3], Jacinto Sá [3,4], Raffaele Marotta [1], Ilaria Di Somma [5], Roberto Andreozzi [1] and Giuseppina Luciani [1]**

1 Department of Chemical, Materials and Industrial Production Engineering, University of Naples "Federico II", p.le V. Tecchio 80, 80125 Napoli, Italy
2 CSGI, Center for Colloid and Interface Science, via della Lastruccia 3, 50019 Sesto Fiorentino, Italy
3 Department of Chemistry-Ångström, Physical Chemistry Division, Uppsala University, P.O. Box 532, 751 20 Uppsala, Sweden
4 Institute of Physical Chemistry, Polish Academy of Sciences, Kasprzaka 44/52, 01-224 Warsaw, Poland
5 Istituto di Scienze e Tecnologie per l'Energia e la Mobilità Sostenibili (STEMS)-Consiglio Nazionale delle Ricerche, p.le V. Tecchio 80, 80125 Napoli, Italy
* Correspondence: laura.clarizia2@unina.it (L.C.); giuseppe.vitiello@unina.it (G.V.)

**Table S1.** Effective radiative powers of the medium-pressure mercury lamp (Helios Italquartz).

| **λ (nm)** | **Effective radiative power (Ein/s)** |
|---|---|
| 305 | 2,23E-06 |
| 313 | 2,76E-06 |
| 366 | 3,37E-06 |
| 404,7 | 8,88E-07 |
| 407,8 | 2,01E-07 |
| 435,8 | 1,47E-06 |
| 546,1 | 1,65E-06 |

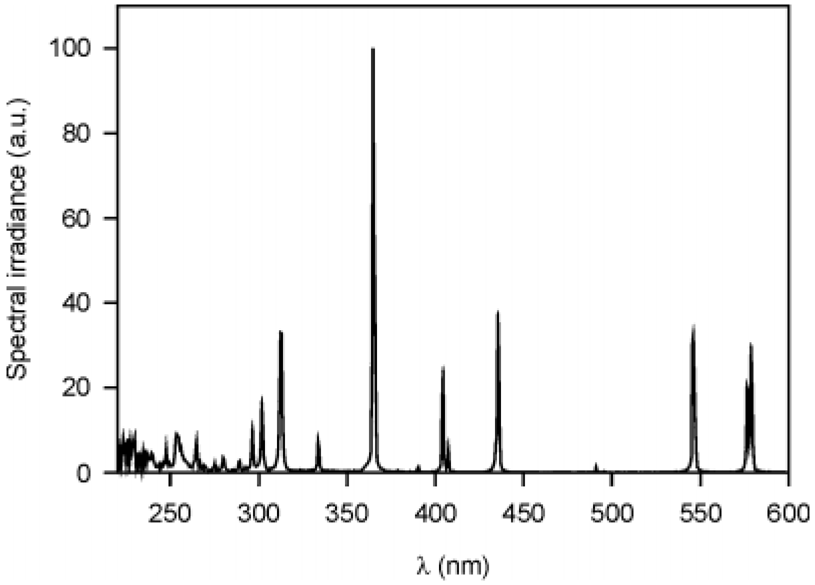


**Figure S1.** The emission spectrum of a medium-pressure mercury lamp Helios Italquartz 125W.

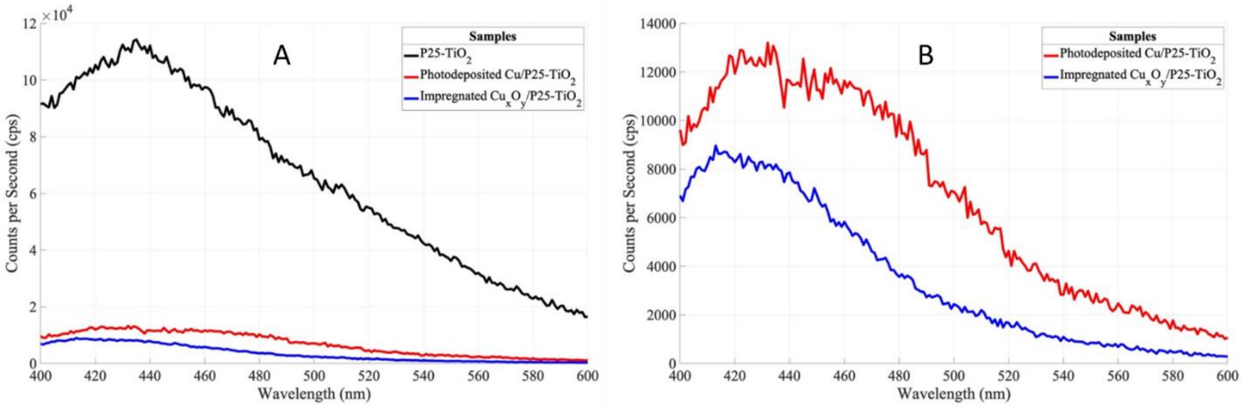


**Figure S2.** PL spectra were measured in degassed pH 4 aqueous solution. P25-$TiO_2$_NPs (black trace). $Cu_xO_y$/$TiO_2$-P25_NPs (blue trace) and Cu/$TiO_2$-P25_NPs (red trace).
**(A)** all the samples; and (**B**) only copper-modified samples.

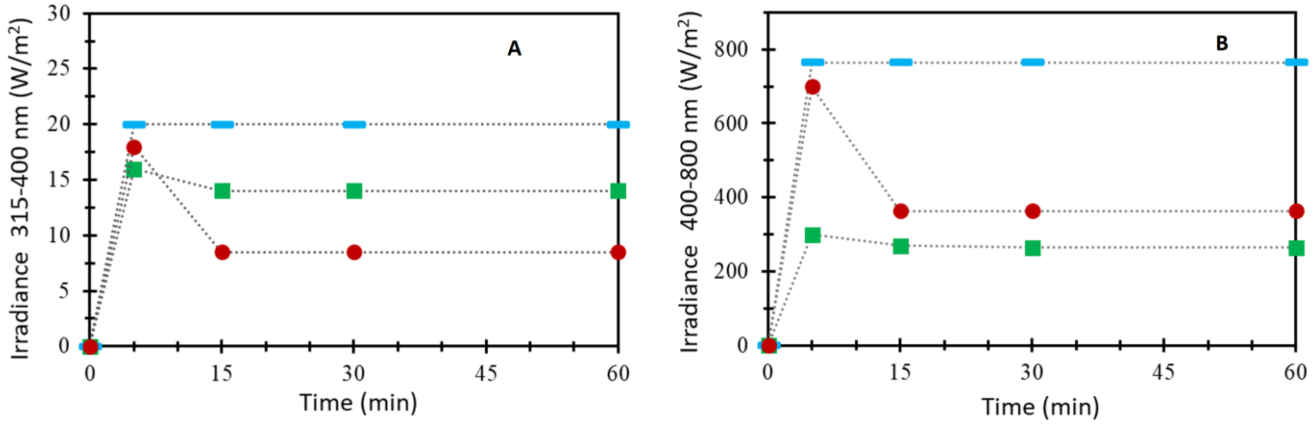


**Figure S3.** Irradiance values measured between **(A)** 315–400 nm and **(B)** 400–800 nm for during methanol photoreforming under de-aerated conditions in the presence of Cu/$TiO_2$_NPs prepared via photodeposition (●), $Cu_xO_y/TiO_2$_NPs prepared via impregnation method (■), and bare P25-$TiO_2$(—).
Copper/$TiO_2$ weight ratio=3%; $[CH_3OH]_0$= 2.47 M; photocatalyst load= 150 mg·$L^{-1}$; T=25 °C; P=1 atm.

## Information on the iterative optimisation procedure developed

Values for the unknown parameters ($k_r$, $N$, and $\Phi_{UV}$) were estimated by an iterative optimisation procedure (Marquardt approach) that minimised the square of the differences between calculated and measured data for hydrogen generation rate (i.e., objective function)[ Reklaitis GV, Ravindran A, Regsdell KM. Engineering Optimization. Wiley, New York, 1983]:

$$\phi = \sum_{l=1}^{m}\sum_{j=1}^{n}\sum_{i=1}^{h}\left(y_{i,j,l} - c_{ijl}\right)^2$$

in which the terms $y$ and $c$ are the calculated and experimental concentrations, where m, n, and h are the number of experimental data recorded in each experiment, the number of the involved species and the number of experiments used in the optimisation procedure, respectively.
